# Associations of metabolic disorder factors with the risk of uncontrolled hypertension: a follow-up cohort in rural China

**DOI:** 10.1038/s41598-017-00789-2

**Published:** 2017-04-07

**Authors:** Jing Xiao, Tianqi Hua, Huan Shen, Min Zhang, Xiao-Jian Wang, Yue-Xia Gao, Qinyun Lu, Chuanli Wu

**Affiliations:** 1 Department of Epidemiology and Medical Statistics, School of Public Health, Nantong University, Nantong, Jiangsu 226019 P.R. China; 2 Department of Chronic Disease and Prevention, Center for Disease Control and Prevention of Haian, Nantong, Jiangsu 226600 P.R. China

## Abstract

We evaluated how metabolic disorders affected antihypertension therapy. 2,912 rural Chinese patients with hypertension who provided blood samples, demographic and clinical data at baseline and after 1 year of antihypertension therapy were evaluated. At baseline, 1,515 patients (52.0%) were already receiving drug therapy and 11.4% of them had controlled blood pressure (BP). After 1 year, all 2,912 patients were receiving antihypertension therapy that was administered by community physicians, and 59.42% of them had controlled BP. Central obesity and abnormal triglyceride, high-density lipoprotein cholesterol, and glucose were associated with 15–70% higher risks of uncontrolled hypertension. Metabolic syndrome using the JIS criteria was associated with poor BP control (odds ratio: 1.71 and 1.54 for the baseline and follow-up datasets, respectively). The risk of uncontrolled hypertension increased with the number of metabolic disorders (p for trend <0.01). The presence of ≥3 metabolic disorder factors was associated with higher risks of poor BP control. The associations of metabolic factors and uncontrolled hypertension were stronger for the standard and modified ATP III criteria, compared to the IDF and JIS criteria. Metabolic factors were associated with less effective antihypertension therapy, and all definitions of metabolic syndrome helped identify patients with elevated risks of uncontrolled hypertension.

## Introduction

Hypertension is a major independent risk factor for cardiovascular disease (CVD)^1^. Although antihypertensive drug treatment has significantly reduced cardiovascular morbidity and mortality^2^, the treated individuals still have considerable risks of CVD and elevated blood pressure (BP)^3^. In this context, hypertension is a metabolic risk factor that is combined with abdominal obesity, dyslipidemia, and hyperglycemia to define metabolic syndrome (MS)^4^. Thus, patients with hypertension have a higher risk of developing MS, and their BP is difficult to control^5^. Furthermore, it has been reported that the incidence of hypertension can be predicted by MS^6^. In addition, some studies have revealed that BP control is significantly reduced in patients with hypertension and MS^7, 8^ or diabetes mellitus^7, 9^, and that BP control worsens when more metabolic disorder factors are present^5^. Moreover, several other studies have revealed that MS and its individual metabolic disorder factors are associated with higher BP, and can affect BP control, among patients who are receiving antihypertension therapy^10, 11^. However, the associations and effect sizes vary according to the definition of MS^12^, which has not been standardized. Previous research has also revealed the need for additional studies regarding whether metabolic disorders can affect BP control among patients receiving antihypertension therapy^5, 7, 8, 13^, and no studies have focused on the association of metabolic disorder factors with poor BP control in rural China.

During 2011, the Haian Hypertension Patients Intervention Study (HHPIS) was launched in Haian (rural China), and enrolled 12,892 individuals during March–May 2011. A total of 2,912 individuals with hypertension (1,211 men and 1,701 women) were included in the present study, which aimed to estimate the BP control rate after antihypertension therapy and its associations with MS and metabolic disorder factors. We also compared the results according to four MS criteria: the Adult Treatment Panel III (ATP III; part of the 2001 US Third Report of the National Cholesterol Education Program)^14^, the 2004 modification of the ATP III for Asian populations (modified ATP III)^15^, the 2005 criteria from the International Diabetes Federation (IDF)^16^, and the 2009 criteria from the Joint Interim Statement (JIS)^17^.

## Methods

### Study population

The HPPIS recruited 12,892 volunteers (18–75 years old) from six Haian county communities with similar economic statuses. All participants were evaluated through in-person interviews during March–May 2011, and 3,082 adults with hypertension were identified at baseline (untreated BP of >140/90 mmHg or receiving antihypertension drug therapy). The exclusion criteria were individuals who were currently participating in a drug trial or had severe psychiatric or neurological illnesses, heart failure, hemodynamically significant valvular disease, unstable coronary heart disease, chronic kidney disease, or the clinical conditions that could affect the diagnosis of MS (e.g., thyroid dysfunction). The present study used a pre-post design to evaluate the intervention cohort, with measurements at baseline and the 1-year follow-up. At baseline, 3,014 patients with hypertension (response rate: 97.79%) completed questionnaires and volunteered to receive an antihypertension intervention treatment (described in detail in the following section). At the 1-year follow-ups, 2,912 patients with hypertension completed questionnaires (response rate: 96.62%). Among these 2,912 patients, 1,515 patients (52.03%) were receiving drug therapy at baseline, and all 2,912 patients had received the antihypertension intervention therapy for 1 year. The total response rate was 94.48%. The study protocol was reviewed and approved by the Board of Scientific Research at Nantong University and the ethical committee of the Haian Center for Disease Control and Prevention. All participants provided their written informed consent, and the study’s methods complied with the approved guidelines and regulations.

### Pharmaceutical and lifestyle intervention

The 2,912 patients with hypertension were divided into two groups based on the type of antihypertension therapy administered for 1 year: 967 patients from two Haian county communities who only received drug therapy and 1,945 patients from four Haian county communities who received drug + lifestyle intervention therapy. Community physicians administered both therapies and attended two 60-min lectures and three 30-min group sessions that were held by the study investigators, which addressed optimal pharmaceutical therapy and lifestyle interventions for treating hypertension. After the baseline data collection, the drug therapy was administered in the form of targeted drugs and dosages that were selected by community physicians based on each patient’s BP (all 2,912 hypertension cases). The community physicians also monitored medication compliance and the patient’s BP through regular follow-ups (twice per month), and provided appropriate medical advice as necessary. The 1,945 patients who also received the lifestyle intervention therapy attended two 60-min individual counseling sessions (4 weeks apart) and a 60-minute group session (20 participants) after 4 weeks, which were all administered by the community physicians. All participants received written instructions to eat foods with low salt contents; increase their intake of vegetables, fruits, and milk; eat white meat instead of red meat; eat fish at 1–2 meals per week; perform moderate exercise (e.g., jogging and dancing) or vigorous exercise (e.g., playing basketball or badminton) for ≥3 h per week; and quit smoking and drinking. The implementation of these instructions was monitored by the community physicians during regular follow-ups (twice per month). All participants provided information regarding their lifestyle factors, anthropometric measurements, and clinical history. Each participant was evaluated at baseline and after 1 year of the antihypertension intervention therapy.

### Anthropometric and biochemical measurements

During the in-person interviews at baseline, each participant’s height was measured twice to prevent reading and typing errors. Weight and waist circumference were also measured twice at the baseline and 1-year follow-up. A third measurement was taken if the difference between the two measurements was >1 cm or >1 kg. The two closest values for height, weight, and waist circumference were averaged and used for the analysis. The average coefficients for intra-observer variation were <0.2% for the height, weight, and waist circumference measurements. Body mass index (BMI) was calculated as weight divided by height squared (kg/m^2^); obesity was defined as a BMI of >28 kg/m^2^, overweight was defined as a BMI of 24–28 kg/m^2^, and normal weight was defined as a BMI of <24 kg/m^2^.

Systolic and diastolic BP were measured three times at baseline and the 1-year follow-up using a standardized mercury sphygmomanometer on the right upper arm. The patient rested for ≥5 min between each measurement, and the average of the three measurements was used for the analysis. Cases of uncontrolled hypertension were defined based on a BP of >140/90 mmHg despite receiving antihypertension therapy. Fasting blood samples were also obtained at baseline and the 1-year follow-up. The 10-mL samples were collected into vacutainers with ethylenediaminetetraacetic acid and stored in a portable Styrofoam box with ice packs before the analysis. Serum levels of triglycerides, high-density lipoprotein cholesterol (HDL-c), and glucose were measured using automated standardized enzymatic methods.

### Lifestyle factors and disease history

Smokers were defined as patients who had smoked at least one cigarette per day during the last month. Drinkers were defined as patients who consumed at least >0.5 oz of ethanol per week during the last month (4 oz of grape wine, 4.8 oz of rice wine, 12 oz of beer, or 1 oz of liquor)^18^. Patients were questioned regarding their familial history of hypertension. We also evaluated the frequency at which they consumed fruit and vegetables (daily, weekly, monthly, yearly, or never) at the 1-year follow-up, as well as the amount consumed in liang (1 liang = 50 g) per unit of time.

### Definitions of metabolic syndrome

The ATP III criteria^14^: patients with hypertension were classified as having MS if they had ≥2 of the following components: waist circumference of ≥102 cm (men) or ≥88 cm (women), serum triglyceride levels of >1.70 mmol/L, receiving lipid-related medication, HDL-c levels of <1.04 mmol/L (men) or <1.29 mmol/L (women), serum glucose levels of ≥6.1 mmol/L, or receiving diabetes medication.

The modified ATP III criteria^15^: patients with hypertension were classified as having MS if they had ≥2 of the ATP III components, but using ethnicity-specific cut-offs for waist circumference (≥90 cm for Chinese men or ≥88 cm for Chinese women) and fasting glucose (≥5.6 mmol/L).

The 2005 IDF criteria^16^: patients with hypertension were classified as having MS if they exhibited central obesity (waist circumference of ≥90 cm for men or ≥80 cm for women) plus one of the following risk factors: serum triglyceride levels of >1.70 mmol/L, receiving lipid-related medication, serum HDL-c levels of <1.04 mmol/L (men) or <1.29 mmol/L (women), serum glucose levels of ≥5.6 mmol/L, or receiving diabetes medication.

The JIS criteria^17^: patients with hypertension were classified as having MS if they had ≥2 of the following components: waist circumference of ≥85 cm (Chinese men) or ≥80 cm (Chinese women), fasting serum triglyceride levels of ≥1.7 mmol/L, receiving lipid-related medication, fasting serum HDL-c levels of <1.0 mmol/L (Chinese men) or <1.3 mmol/L (Chinese women), receiving specific treatment for elevated HDL-c levels, serum glucose levels of ≥5.6 mmol/L, or receiving diabetes medication.

### Statistical analysis

Demographic, dietary, lifestyle, and biochemical characteristics were reported as mean ± standard deviation (continuous variables) or as number and percentage (categorical variables). Differences were evaluated using analysis of ANOVA (continuous variables) or the chi-square test (categorical variables). Odds ratios (ORs) and 95% confidence intervals (95% CIs) were estimated using non-conditional logistic regression analyses to evaluate the associations of metabolic disorder factors with the risk of uncontrolled hypertension at baseline. Conditional logistic regression analyses with condition on two antihypertension intervention therapies were used to evaluate the associations of metabolic disorder factors with the risk of uncontrolled hypertension at the follow-up. The regression models were all adjusted for potential confounders (i.e., age at interview (a continuous variable), occupation, smoking status, and drinking status). All statistical analyses were performed using SAS software (version 9.2; SAS Institute, Cary, NC), all tests were two-sided, and differences were considered statistically significant at P-values of <0.05.

## Results

Among the 3,082 participants with hypertension in the HPPIS, we received completed questionnaires from 2,912 patients at the 1-year follow-up. Among these 2,912 patients, 1,515 patients (52.03%) were receiving drug therapy at baseline and all 2,912 patients were receiving the antihypertension intervention therapy at the 1-year follow-up. The rates of medication compliance were 48.09% and 77.51% at 1 month and 1 year, respectively. Better BP control was observed at the follow-up, compared to baseline (59.42% vs. 11.40%, respectively). At baseline, 22.63% of the patients had BP values of 160–179/100–109 mmHg and 5.34% of the patients had BP values of ≥180/110 mmHg. At the 1-year follow-up, 7.95% of the patients had BP values of 160–179/100–109 mmHg and 2.74% of the patients had BP values of ≥180/110 mmHg. The detailed distributions of the hypertension grades are presented in Table 1. Table 2 presents the prevalences of MS according to the four MS criteria among the patients with hypertension. The prevalences of MS were higher at baseline for all criteria, compared to after 1 year of antihypertension therapy.

**Table 1:** Patient distributions according to hypertension grade.

	Optimal	Normal	Grade I	Grade II	Grade III
Systolic blood pressure (mmHg)	<130	130–139	140–159	160–179	≥180
Diastolic blood pressure (mmHg)	<80	80–89	90–99	100–109	≥110
Prevalence at baseline (%)	5.43	5.97	60.63	22.63	5.34
Prevalence at the follow-up (%)	5.75	53.67	29.89	7.95	2.74

**Table 2:** The prevalences of MS according to four MS criteria among patients with hypertension.

MS prevalence	ATP III	Modified ATP III	IDF	JIS
Baseline	57.60%	74.62%	62.80%	84.41%
Follow-up	35.37%	51.10%	49.66%	65.04%

MS: metabolic syndrome, ATP III: the Adult Treatment Panel III (part of the 2001 US Third Report of the National Cholesterol Education Program), Modified ATP III: the 2004 modification of the ATP III for Asian populations, IDF: the 2005 criteria from the International Diabetes Federation, JIS: the 2009 criteria from the Joint Interim Statement.

Table 3 lists the proportion of controlled and uncontrolled BP for patients with hypertension in the MS and non-MS groups at baseline and follow-up. Compared to patients without MS, patients with MS had higher rates of uncontrolled BP at baseline (91.56% vs. 72.46%) and at the follow-up (45.35% vs. 31.83%). The same difference at the follow-up was observed among patients who received only drug therapy (66.40% vs. 57.52%) and patients who received drug + lifestyle intervention therapy (34.91% vs. 19.00%). Moreover, the follow-up rates of uncontrolled BP after only drug therapy (with MS: 66.40%, without MS: 57.25%) were much higher than after drug + lifestyle intervention therapy (with MS: 34.91%, without MS: 19.00%).

**Table 3:** The proportion of controlled and uncontrolled BP for patients with hypertension in the MS (JIS criteria) and non-MS groups at baseline and the 1-year follow-up.

Treatments		MS		Non-MS		P
		Controlled BP	Uncontrolled BP	Controlled BP	Uncontrolled BP	
Baseline	Drug (%)	108 (8.44)	1,171 (91.56)	65 (27.54)	171 (72.46)	<0.01
Follow-up	Group one^1^ (%)	211 (33.60)	417 (66.40)	144 (42.48)	195 (57.52)	0.02
	Group two^2^ (%)	824 (65.09)	442 (34.91)	550 (81.00)	129 (19.00)	<0.01
	Total (%)	1,035 (54.65)	859 (45.35)	694 (68.17)	323 (31.83)	<0.01

^1^Only drug therapy, ^2^Drug + lifestyle intervention therapy.

BP: blood pressure, MS: metabolic syndrome, JIS: the 2009 criteria from the Joint Interim Statement.

The patient characteristics according to controlled or uncontrolled hypertension are summarized in Table 4. The two groups had similar values for age, sex, BMI, income, education level, and familial history of hypertension (all, P > 0.05). However, compared to the group with controlled hypertension, the group with uncontrolled hypertension had higher values for waist circumference, triglycerides, and glucose; lower values for HDL-c; less vegetable and fruit consumption; and greater prevalences of smoking and drinking (all, P < 0.01). The group with uncontrolled hypertension was more likely to have MS, based all four MS criteria. Uncontrolled hypertension was significantly more common among farmers at baseline (P < 0.01), although a non-farming occupation was associated with uncontrolled hypertension at the 1-year follow-up. In addition, patients with controlled hypertension were more likely to receive drug + lifestyle intervention therapy, compared to patients with uncontrolled hypertension.

**Table 4:** Characteristics of cases with controlled or uncontrolled hypertension among patients receiving antihypertension therapy.

Variable	Baseline^*a*^		***P***	1-year follow-up^*b*^		***P***
	Controlled cases	Uncontrolled cases		Controlled cases	Uncontrolled cases	
Age (years)	58.87 ± 9.13	58.10 ± 7.66	0.25	57.62 ± 7.88	58.16 ± 8.13	0.08
BMI (kg/m^2^)	26.16 ± 3.56	26.62 ± 3.53	0.08	24.93 ± 3.97	25.19 ± 3.94	0.08
WC (cm)	84.96 ± 10.66	88.72 ± 9.44	<0.01	86.16 ± 9.10	88.10 ± 10.16	<0.01
Vegetables (kg/day)	0.46 ± 0.13	0.39 ± 0.16	<0.01	0.51 ± 0.23	0.45 ± 0.15	<0.01
Fruits (kg/day)	0.17 ± 0.07	0.08 ± 0.08	<0.01	0.16 ± 0.10	0.10 ± 0.06	<0.01
TG (mmol/L)	2.31 ± 1.47	2.62 ± 1.64	<0.01	1.85 ± 1.24	2.09 ± 1.39	<0.01
HDL-c (mmol/L)	1.70 ± 0.62	1.57 ± 0.59	<0.01	1.61 ± 0.35	1.48 ± 0.40	<0.01
Glucose (mmol/L)	6.14 ± 1.74	6.50 ± 1.80	<0.01	5.90 ± 2.03	6.17 ± 2.27	<0.01
Sex						
Female	110 (62.50)	775 (57.88)		1,034 (59.80)	667 (56.38)	
Male	66 (37.50)	564 (42.12)	0.24	695 (40.20)	516 (43.62)	0.07
Education						
≤Primary school	156 (88.64)	1,143 (85.36)		1,489 (86.12)	1,036 (87.57)	
Middle school	11 (6.25)	149 (11.13)		181 (10.47)	112 (9.47)	
≥High school	9 (5.11)	47 (3.51)	0.09	59 (3.41)	35 (2.96)	0.52
Income/month						
≤500 yuan	9 (5.34)	119 (8.89)		146 (8.45)	104 (8.79)	
500–1,000 yuan	15 (8.40)	130 (9.71)		169 (9.77)	109 (9.21)	
≥1,000 yuan	152 (86.26)	1,090 (81.40)	0.19	1,414 (81.78)	970 (81.99)	0.85
Farmer						
No	112 (63.64)	610 (45.56)		744 (43.03)	731 (61.79)	
Yes	64 (36.36)	729 (54.44)	<0.01	985 (56.97)	452 (38.21)	<0.01
Ever smoked						
No	164 (93.18)	1,062 (79.31)		1,460 (84.44)	944 (79.80)	
Yes	12 (6.82)	277 (20.69)	<0.01	269 (15.56)	239 (20.20)	<0.01
Ever drinker						
No	161 (91.48)	1,035 (77.30)		1,476 (85.37)	915 (77.35)	
Yes	15 (8.52)	304 (22.70)	<0.01	253 (14.63)	268 (22.65)	<0.01
Familial history of hypertension						
No	141 (80.11)	1,096 (81.85)		1,435 (83.00)	976 (82.50)	
Yes	35 (19.89)	243 (18.15)	0.58	294 (17.00)	207 (17.50)	0.73
Having MS (IDF)						
No	95 (53.98)	550 (41.08)		909 (52.57)	557 (47.08)	
Yes	81 (46.02)	789 (58.92)	<0.01	820 (47.43)	626 (52.92)	<0.01
Having MS (ATP III)						
No	106 (60.31)	543 (40.55)		1,167 (67.50)	715 (60.44)	
Yes	70 (39.77)	796 (59.45)	<0.01	562 (32.50)	468 (39.56)	<0.01
Having MS (modified ATP III)						
No	86 (48.86)	381 (28.45)		908 (52.52)	516 (43.62)	
Yes	90 (51.14)	958 (71.55)	<0.01	821 (47.48)	667 (56.38)	<0.01
Having MS (JIS)						
No	56 (32.06)	188 (14.04)		651 (37.65)	367 (31.02)	
Yes	120 (68.18)	1,151 (85.96)	<0.01	1,078 (62.35)	816 (68.98)	<0.01
Means of intervention						
Drug	—	—		355 (20.53)	612 (51.73)	
Drug + lifestyle	—	—	—	1,374 (79.46)	571 (48.27)	<0.01

WC: waist circumference, TG: triglycerides, HDL-c: high-density lipoprotein cholesterol, MS: metabolic syndrome, ATP III: the Adult Treatment Panel III (part of the 2001 US Third Report of the National Cholesterol Education Program), Modified ATP III: the 2004 modification of the ATP III for Asian populations, IDF: the 2005 criteria from the International Diabetes Federation, JIS: the 2009 criteria from the Joint Interim Statement.

^*a*^1,515 patients with hypertension who received drug therapy among the 2,912 patients at baseline, ^*b*^2,912 patients with hypertension who were all receiving antihypertension therapy at the 1-year follow-up.

Data are shown as mean ± standard deviation or number (%).

The associations of MS and metabolic disorder factors with uncontrolled hypertension were evaluated after adjusting for potential confounders using the baseline and follow-up datasets separately (Table 5). All four metabolic risk factors (central obesity and abnormal values for triglycerides, HDL-c, and glucose) were associated with 15–70% higher risks of uncontrolled hypertension, according to all four MS criteria. In addition, based on the ATP criteria, MS was the strongest independent and treatable predictor of uncontrolled BP (baseline, OR: 2.02, 95% CI: 1.12–3.09; follow-up, OR: 1.60, 95% CI: 1.28–1.96). Furthermore, the risk of uncontrolled hypertension increased with the number of metabolic disorder factors (P for trend < 0.01). However, the magnitude of the effect significantly depended on the definition that was used, and the presence of ≥3 metabolic disorder factors from the standard and modified ATP III criteria provided higher risks of poor BP control (standard criteria: 210% at baseline and 221% at follow-up, modified criteria: 207% at baseline and 197% at follow-up). Similar results were observed for the baseline and 1-year follow-up datasets.

**Table 5:** Association of uncontrolled hypertension and metabolic risk factors among hypertension patients from baseline and the follow-up^*^.

Variable	ATP III MS definition		Modified ATP III MS definition		IDF MS definition		JIS MS definition	
	OR (95% CI)^*a*^	OR (95% CI)^*b*^	OR (95% CI)^*a*^	OR (95% CI)^*b*^	OR (95% CI)^*a*^	OR (95% CI)^*b*^	OR (95% CI)^*a*^	OR (95% CI)^*b*^
Central obesity vs. normal waist circumference^*c*^	1.57 (1.13–2.46)	1.41 (1.17–1.71)	1.68 (1.21–2.60)	1.46 (1.23–1.72)	1.65 (1.25–3.18)	1.30 (1.10–1.55)	1.56 (1.14–3.78)	1.28 (1.07–1.53)
Abnormal vs. normal TG	1.21 (1.08–1.74)	1.32 (1.11–1.56)	1.21 (1.08–1.74)	1.32 (1.11–1.56)	1.21 (1.08–1.74)	1.32 (1.11–1.56)	1.19 (1.06–1.69)	1.32 (1.11–1.56)
Abnormal vs. normal HDL-c	1.17 (1.07–2.34)	1.21 (1.07–1.41)	1.17 (1.07–2.34)	1.21 (1.07–1.41)	1.17 (1.07–2.34)	1.21 (1.07–1.41)	1.16 (1.04–2.80)	1.19 (1.05–1.38)
Abnormal vs. normal glucose	1.39 (1.11–2.11)	1.31 (1.11–1.55)	1.28 (1.04–1.77)	1.38 (1.15–1.65)	1.28 (1.04–1.77)	1.38 (1.15–1.65)	1.28 (1.04–1.77)	1.38 (1.15–1.65)
MS	2.02 (1.12–3.09)	1.60 (1.28–1.96)	1.96 (1.13–3.00)	1.60 (1.37–1.97)	1.76 (1.48–2.39)	1.55 (1.15–1.90)	1.71 (1.16–2.82)	1.54 (1.13–1.90)
Metabolic disorder factors								
0	1.0	1.0	1.0	1.0	1.0	1.0	1.0	1.0
1	1.41 (1.01–2.82)	1.19 (0.91–1.56)	1.47 (1.04–2.67)	1.19 (0.91–1.56)	1.41 (1.17–3.38)	1.24 (1.00–1.59)	1.56 (0.91–2.76)	1.28 (1.02–2.04)
2	2.15 (1.34–3.37)	1.87 (1.01–2.20)	2.03 (1.28–3.17)	1.89 (1.43–2.26)	1.66 (1.04–2.64)	1.42 (1.09–1.61)	2.11 (1.44–3.74)	1.98 (1.43–2.41)
≥3	3.10 (2.23–3.78)	3.21 (2.65–3.97)	3.07 (2.67–3.98)	2.97 (2.35–3.57)	2.57 (1.29–3.13)	2.41 (1.72–2.78)	2.68 (1.64–3.66)	2.34 (1.76–3.05)
P for trend	<0.01	<0.01	<0.01	<0.01	<0.01	<0.01	<0.01	<0.01
Area under the receiver operating characteristic curve (95% CI)	0.63 (0.56–0.69)	0.56 (0.54–0.58)	0.62 (0.55–0.68)	0.57 (0.54–0.59)	0.61 (0.55–0.68)	0.55 (0.53–0.57)	0.63 (0.56–0.70)	0.56 (0.53–0.58)

^*^Adjusted for body mass index (not including in model *c*), vegetable consumption, fruit consumption, education, income, farmer status, smoking status and drinking status.

^a^1,515 patients with hypertension who received drug therapy among the 2,912 patients at baseline, ^*b*^2,912 patients with hypertension who were all receiving antihypertension therapy at the 1-year follow-up.

OR: odds ratio, 95% CI: 95% confidence interval, TG: triglycerides; HDL-c: high-density lipoprotein cholesterol, MS: metabolic syndrome, ATP III: the Adult Treatment Panel III (part of the 2001 US Third Report of the National Cholesterol Education Program), Modified ATP III: the 2004 modification of the ATP III for Asian populations, IDF: the 2005 criteria from the International Diabetes Federation, JIS: the 2009 criteria from the Joint Interim Statement.

## Discussion

The present study revealed that MS and the four metabolic disorder factors decreased the probability of BP control among patients who were receiving antihypertension treatment. Furthermore, the risk of uncontrolled hypertension was associated with the number of metabolic disorder factors (P for trend <0.01). The presence of ≥3 metabolic factors was associated with higher risks of uncontrolled hypertension that varied (141–221%) according to the MS criteria, although the ATP criteria were associated with the highest risk of poor BP control when four metabolic disorder factors were present. The baseline BP control rate among rural Chinese patients who were being treated for hypertension was very low (11.40%), compared to the rates of control in other regions of China^19–22^, and in European countries or the US^22–26^. However, in the present study, 1 year of the intervention treatment (lifestyle and/or drug interventions) increased the BP control rate to 59.42%, which is higher than the rates from previous reports^21–27^.

The prevalences of MS among patients with hypertension were high in the present study (84.41% at baseline and 65.04% at the 1-year follow-up based on the JIS criteria). Both hypertension and MS are relevant public health concerns, and metabolic risk factors are closely associated with the development of hypertension^28^. The present study revealed that the prevalence of MS (according to all four criteria) was higher among patients with uncontrolled hypertension, compared to patients with controlled hypertension. This result is consistent with previous findings that patients with hypertension and MS have an elevated prevalence of uncontrolled BP^5, 7^.

We also found that BP control was worse among patients with hypertension and MS, compared to patients without MS. In addition, patients with hypertension and MS had a higher rate of uncontrolled BP after receiving antihypertension intervention therapy, compared to patients with hypertension and without MS. These results are consistent with Zidek *et al*.’s findings that MS is associated with both higher BP and a poor response to treatment^10^. Furthermore, Arcucci *et al*. have reported that BP control worsens in the presence of more metabolic disorder factors^5^. Moreover, we found that uncontrolled hypertension was associated with adverse characteristics (high values for triglycerides, waist circumference, and glucose; low values for HDL-c), compared to controlled hypertension. Similarly, previous studies have demonstrated that poor BP control is associated with obesity and abdominal obesity^29–31^, dyslipidemia^32, 33^, and abnormal glucose metabolism^7^, with 37–61% of patients with hypertension having two metabolic risk factors^11, 34^.

In the present study, we confirmed that metabolic disorder factors were associated with the effects of antihypertension therapy. For example, abdominal obesity and abnormal values for triglycerides, HDL-c, and glucose were associated with 15–70% higher risks of uncontrolled hypertension, compared to the normal groups. In this context, it has been suggested that metabolic disorder factors have additive effects on BP control and cardiovascular disease. Similarly, we found that the presence of MS was associated with a 96% higher risk of uncontrolled hypertension, while having three or four metabolic disorder factors (based on the JIS criteria, which are suited for Chinese people) was associated with 168% and 134% higher risks of poor BP control, respectively. Therefore, it appears that there is an interaction between hypertension and metabolic disorder factors, although the mechanisms that are involved in this interaction remain unclear. Nevertheless, strong evidence suggests that insulin resistance and obesity result in hypertension^8^, and it is reasonable to assume that MS may affect uncontrolled hypertension.

This study has several strengths. First, to the best of our knowledge, the present study is the first to evaluate the effects of individual metabolic disorder factors on BP control in rural China. Second, previous studies have used several different definitions to compare the effects of metabolic disorders on BP control, and the present study considered all four definitions. This study also had some limitations. We did not collect data regarding the treatment dose and duration; thus, the effect of different treatments on BP control could not be adjusted for in the logistic regression model. Accordingly, the metabolic disorder factors and demographic variables that were included in the logistic regression model only partially explained the risk of poor BP control because the largest areas under the receiver operative characteristic curve were 0.63 and 0.56 (for the JIS criteria).

## Conclusions

Hypertension is a major risk factor for CVD morbidity and mortality. Unfortunately, only a small group of Chinese patients with hypertension had optimal BP control, despite significant investments in hypertension treatment by the Chinese government. Therefore, it is important to identify factors that may influence the effectiveness of antihypertension treatment. Our data suggest that patients with hypertension and metabolic disorders factors (or even a single metabolic disorder factor) may have a lower probability of achieving BP control, compared to patients with hypertension and no metabolic disorder factors. In addition, it appears that these factors exert an additive effect on the likelihood of poor BP control. Furthermore, MS based on the ATP III and modified ATP III criteria was associated with a higher risk of uncontrolled hypertension, compared to the IDF and JIS criteria. Moreover, the presence of MS strongly affected the efficacy of antihypertension therapy. These findings indicate that these treatments should include both drug treatment and lifestyle interventions to increase the patient’s resistance to metabolic disorders, which may help significantly improve BP control rates.
